# Supplementary material for: Molecular identification and quantification of defect sites in metal-organic frameworks with NMR probe molecules
Source: Nat Commun. 2022 Aug 30;13:5112. doi: 10.1038/s41467-022-32809-9 (PMC9427814; doi:10.1038/s41467-022-32809-9)
Supplement: Supplementary file 1 — Supplementary Information [file 41467_2022_32809_MOESM1_ESM.pdf]

Supplementary Information for

Molecular Identification and Quantification of Defect Sites in Metal-organic Frameworks with NMR Probe Molecules

Jinglin Yin[1,2,†], Zhengzhong Kang[1,†], Yao Fu[1], Weicheng Cao[1], Yiran Wang[1], Hanxi Guan[1], Yu Yin[1], Binbin Chen[1], Xianfeng Yi[3], Wei Chen[3], Wei Shao[4], Yihan Zhu[4], Anmin Zheng[3], Qi Wang[1], Xueqian Kong[1,2,*]

[1] Department of Chemistry, Zhejiang University, Hangzhou 310027, PR China;

[2] Key Laboratory of Excited-State Materials of Zhejiang Province, Zhejiang University, Hangzhou 310027, PR China;

[3] State Key Laboratory of Magnetic Resonance and Atomic and Molecular Physics, National Center for Magnetic Resonance in Wuhan, Wuhan Institute of Physics and Mathematics, Innovation Academy for Precision Measurement Science and Technology, Chinese Academy of Sciences, Wuhan 430071, PR China;

[4] College of Chemical Engineering and State Key Laboratory Breeding Base of Green Chemistry Synthesis Technology, Zhejiang University of Technology, Hangzhou, 310014, China.

[†] These two authors contributed equally.

Corresponding authors:

Xueqian Kong
Email: kxq@zju.edu.cn

This file includes:
Supplementary Methods
Supplementary Figures 1-8
Supplementary Tables 1-3
Supplementary References 1-14

Supplementary Methods

Theoretical calculations

The structure optimization of chemical compounds and the energy calculation of different defective structures were performed by density function theory (DFT) calculations with the B3LYP method in Gaussian 09 package.[1] The LANL2DZ basis set was applied to the zirconium atoms while the 6-31G* (d, p) basis set for C, H, O, and P atoms.[87] The ^{31}P chemical shifts (δ_{X}) were calculated by the following equation:

$$\delta_{\mathrm{X}} = \sigma_{r\mathrm{ef}} - \sigma_{\mathrm{X}}$$

where subscript X represents TMPO or TMP in different adsorption states. σ_{X} is the absolute chemical shielding of chemical compounds calculated by Gauge-invariant atomic orbital (GIAO) method[2,3] in Gaussian 09 package. σ_{ref} represents the absolute chemical shielding of the reference compound, 379.111 for $NH_4H_2PO_4$. We firstly applied this method to calculate the chemical shifts of small compounds to match the results with experimental ^{31}P NMR data. Eventually, the chemical shifts of TMPO/TMP in various adsorption states were calculated with the same method and basis sets. The binding energies (ΔE) of different defect structures in Supplementary Fig. 4 are calculated by following formula:

$$\Delta E = E_{\mathrm{UIO}_{\mathrm{Zr-R}_j}^{\mathrm{Zr-R}_i}} - E_{\mathrm{UIO}_{\mathrm{Zr*}}^{\mathrm{Zr*}}} - E_{\mathrm{R}_i} - E_{\mathrm{R}_j}$$

Here, R_i and R_j represent different defect-associated molecules and E_{R_i} and E_{R_j} correspond to their energies, respectively. $E_{\mathrm{UIO}_{\mathrm{Zr*}}^{\mathrm{Zr*}}}$ is the energy of a single missing-linker defect site without coordinated molecules. $E_{\mathrm{UIO}_{\mathrm{Zr-R}_j}^{\mathrm{Zr-R}_i}}$ is the energy of a single missing-linker defect site with coordinated molecules.

We also applied molecular dynamics (MD) simulations in the GROMACS package (version 5.0.4)[4-6] to obtain the possible adsorption states of probe molecules in defective UiO-66. Defective

UiO-66 were put in water solutions with probe molecules randomly distributed in MOFs. Each simulation group was set for 50 ns running with periodic boundary conditions. The parameters of MOFs were obtained from the work by Q. Yang and coworkers.[7] While the parameters of chemical compounds were carried out in the General Amber Force Field[8] form by Antechamber tool. The charges were obtained by the RESP method.[9] Position restrain was applied to Zr and μ-O atoms while other atoms were free in the system. Water molecule was described by the simple point charge model.[10] The solution system was neutralized by sodium and chloride ions. The time step was set at 2 fs and simulation data were saved every 4 ps. The bond lengths were constrained by linear constraint solver algorithm.[11] The cutoff switching function for the non-bonded van der Waals interaction starts at 1.2 nm and reaches zero at 1.35 nm, while the cut-off distance of the long-range electrostatic interaction was set at 1.2 nm. The long-range electrostatic interaction was calculated by particle mesh Ewald summation method.[12] The temperature was set at 300 K and was maintained through Nose-Hoover thermostat coupling method.[13]

The adsorption structures of probe molecules in MOFs were obtained from MD simulations. Half clusters coordinated by BDCs were used as the models for NMR calculation. The influence of surrounding BDCs on ^{31}P chemical shifts is contained in the conformation of adsorbed molecule.[14] In defective MOFs, the aromatic ring of the BDC linker is replaced with a ghost atom. In ideal MOFs, one carboxylate on BDC is replaced with a ghost atom. The use of ghost atoms would result in with a slight difference of less than 1 ppm on ^{31}P chemical shift. For the simulation of physical adsorption, the model considers all BDC linkers within 0.5 nm of the probe molecule. The cif files for NMR calculation can be downloaded from the supplementary data files.

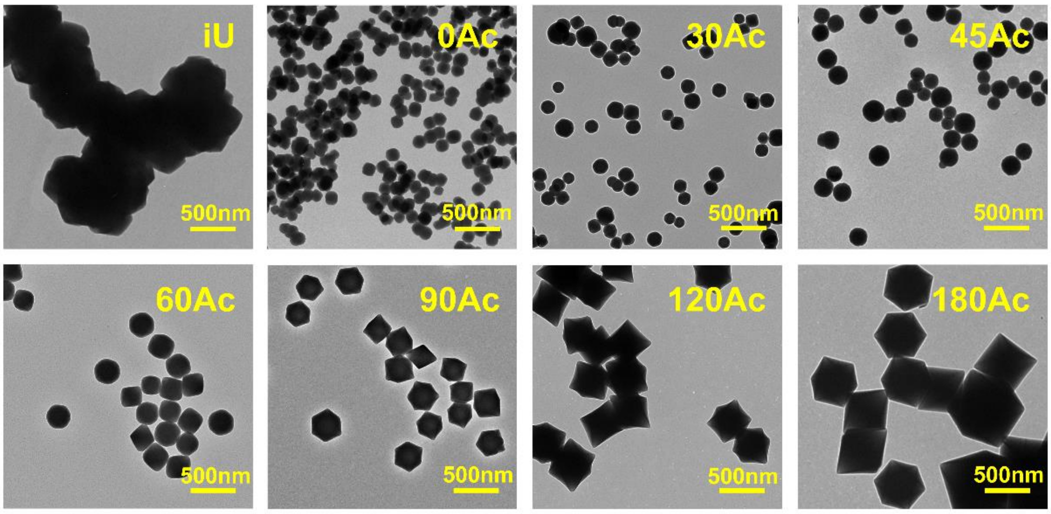

Supplementary Fig. 1. TEM images of ideal (iU) and defective UiO-66 (dU). 0Ac, 30Ac, 45Ac, etc. stand for the molar ratios of acetic acid with respect to the Zr atoms in the synthesis solutions.

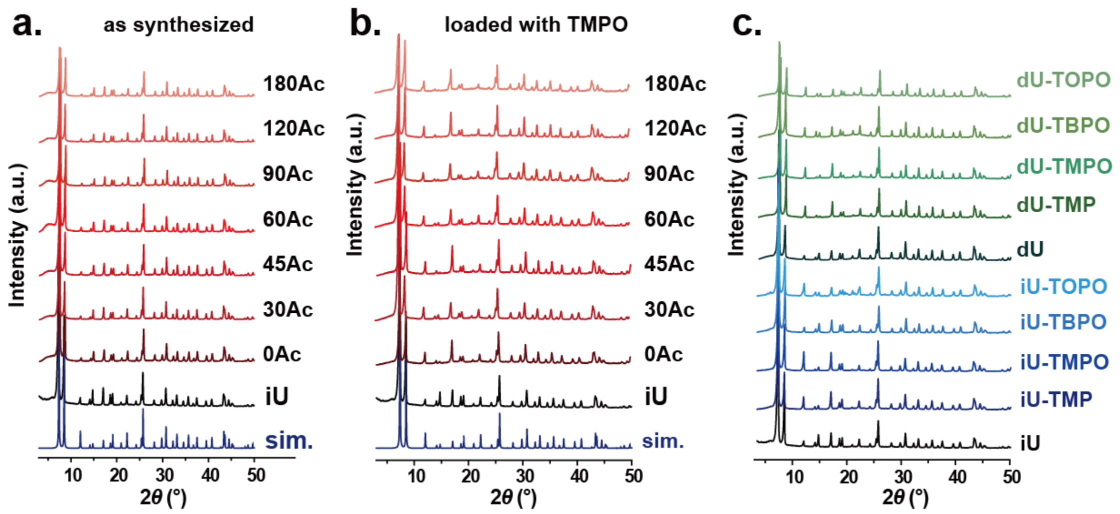

Supplementary Fig. 2. PXRD patterns of ideal (iU) and defective UiO-66 (dU) (**a**) before and (**b, c**) after the adsorption of probe molecules. The crystalline structures of UiO-66 were maintained after the adsorption.

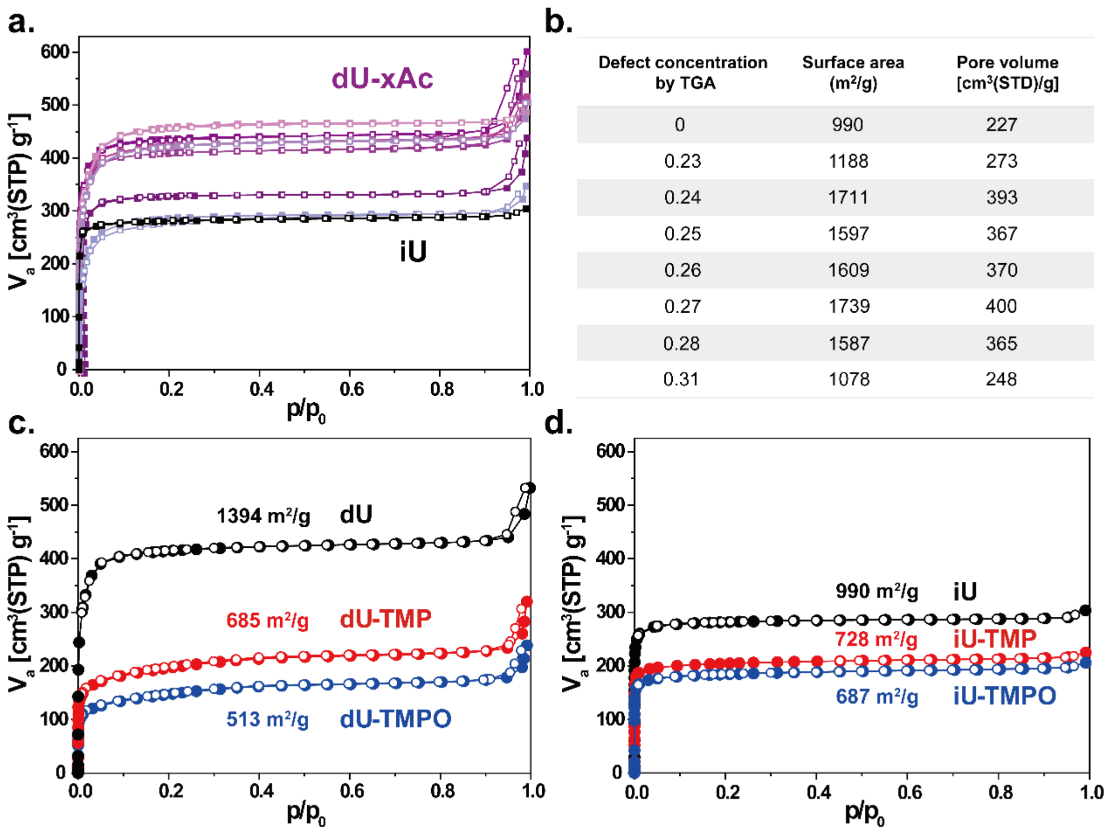

Defect concentration by TGA	Surface area (m^2/g)	Pore volume [cm^3(STD)/g]
0	990	227
0.23	1188	273
0.24	1711	393
0.25	1597	367
0.26	1609	370
0.27	1739	400
0.28	1587	365
0.31	1078	248

Supplementary Fig. 3. (**a**) The nitrogen sorption curves of as-synthesized UiO-66. x stands for the relative concentration of added acetic acid. (**b**) The BET surface areas and pore volume of different samples. The nitrogen sorption curves of (**c**) dU and (**d**) iU before and after the adsorption of probe molecules.

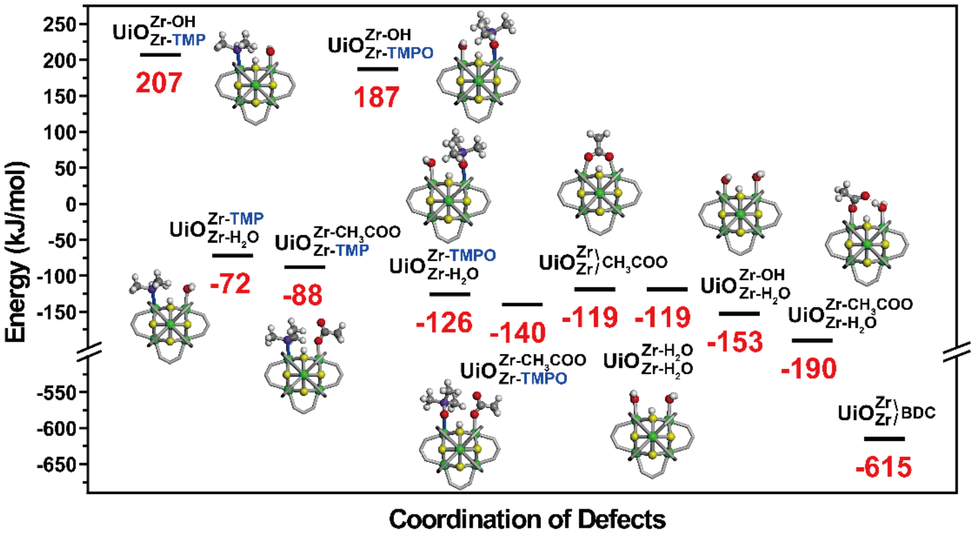

Supplementary Fig. 4. Binding energies of different defect structures. Green spheres for Zr, yellow for μ-O, red for O atoms at defect sites and those of TMPO, gray for C, purple for P, white for H. Lower energy indicates a more stable structure.

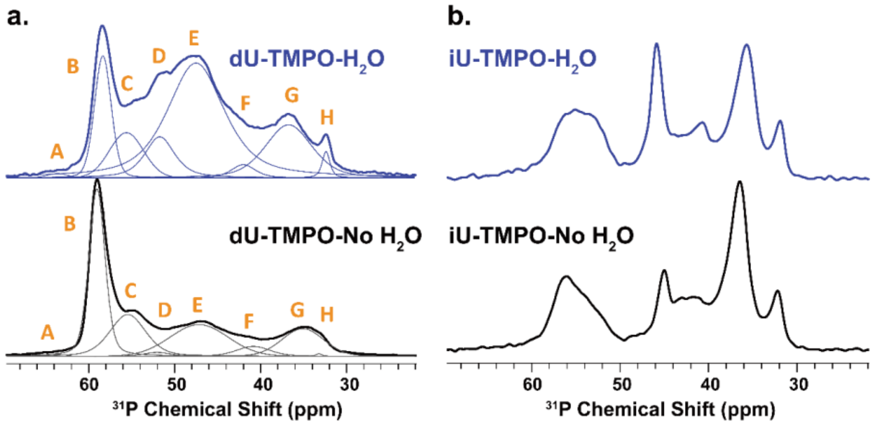

Supplementary Fig. 5. ^{31}P CPMAS spectra of (**a**) dU and (**b**) iU after TMPO adsorption. The black spectra are the samples prepared by avoiding water during adsorption (in the dry box). The blue spectra are the samples prepared with an additional step of immersion in water.

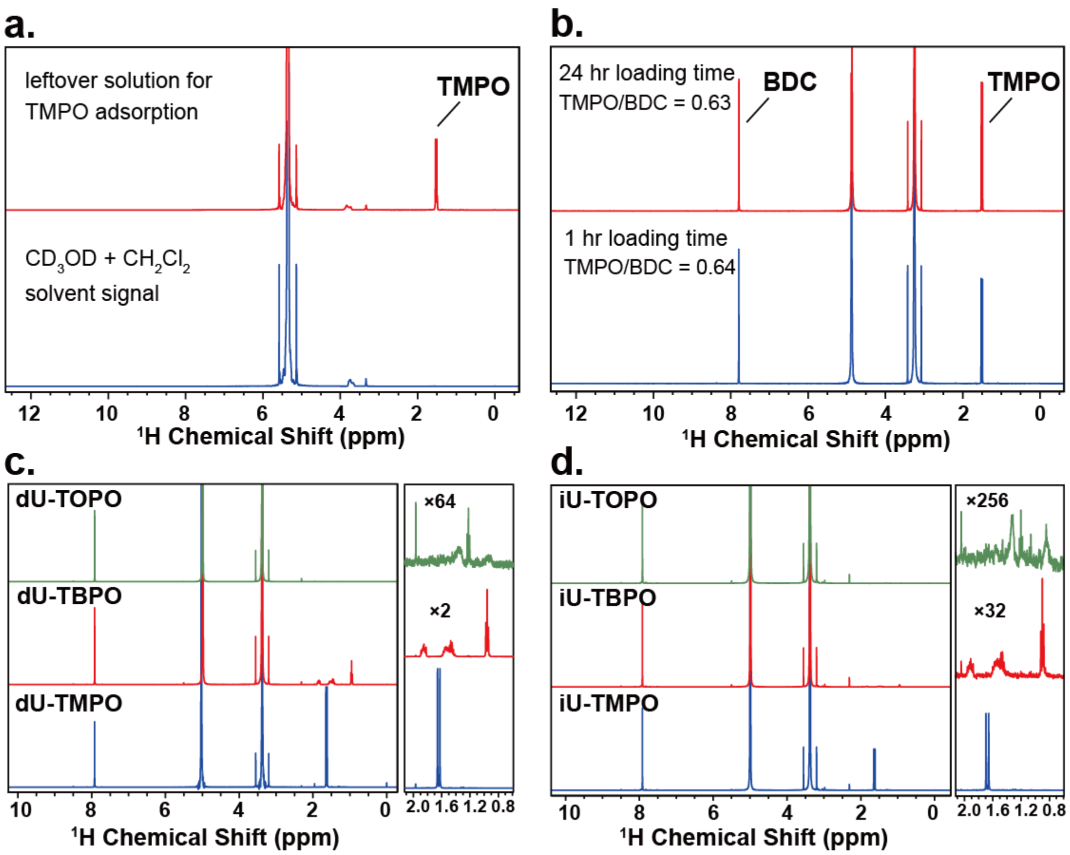

Supplementary Fig. 6. (**a**) ^{1}H solution-state NMR of the leftover TMPO adsorption solution. The TMPO is dissolved in CD_3OD and CH_2Cl_2. There is no signal of the BDC linker or acetic acid in the leftover solution indicating TMPO will not replace chemical species in UiO-66. (**b**) ^{1}H solution-state NMR of NaOH-digested UiO-66 loaded with TMPO. For 1 hr or 24 hrs of adsorption time, the amount of loaded TMPO is the same indicating 1 hr is enough to reach the adsorption equilibrium. ^{1}H solution-state NMR of NaOH-digested (**c**) dU and (**d**) iU loaded with TMPO and its homologs. The integral areas of the methyl hydrogen are used for quantification as shown in the enlarged windows.

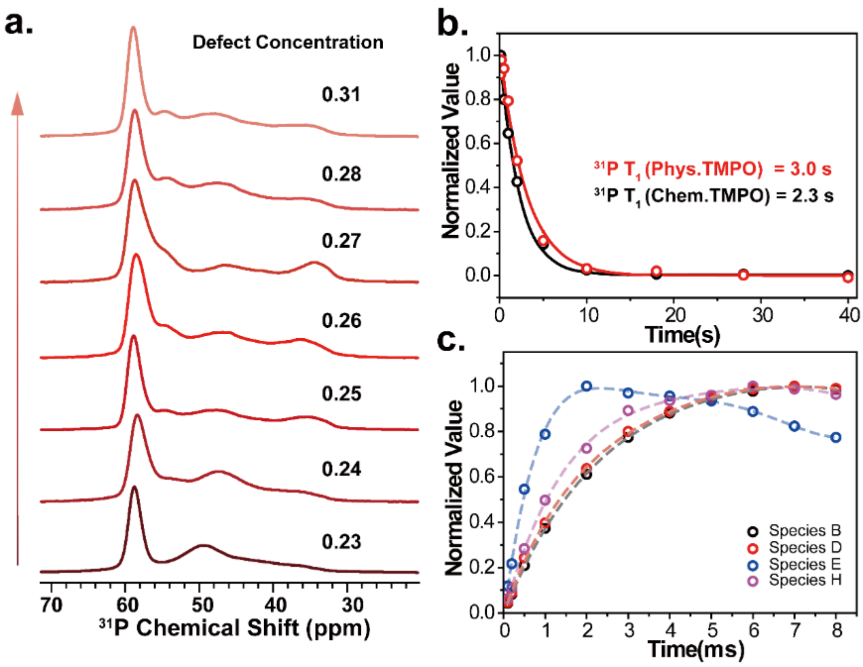

Supplementary Fig. 7. (**a**) ^{31}P direct polarization (DP) spectra of dU loaded with TMPO. The defect concentrations shown on the left were measured by TGA. (**b**) ^{31}P spin-lattice relaxation (T_1) curves of a TMPO-loaded sample. (**c**) ^{1}H-^{31}P cross polarization (CP) curves of different contact times for species B, D, E and H in a TMPO-loaded sample.

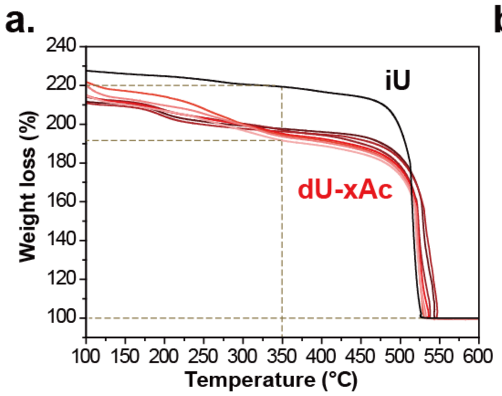

b.

Weight Ratio	x	Defect Concentration	
197.6 %	1.12	0.23	
197.0 %	1.15	0.24	
196.2 %	1.19	0.25	
195.1 %	1.25	0.26	
194.4 %	1.28	0.27	
193.5 %	1.32	0.28	
191.6 %	1.42	0.31	
219.3 %	0.05	0.01	iU

Supplementary Fig. 8. (**a**) The weight loss curves of different UiO-66 samples measured by TGA. The remaining weight at 600°C is set as 100%. The weight at 350°C is considered as the weight of MOF without trapped small molecules. (**b**) The quantified defect concentrations of various samples from the TGA measurement. x represents the value in formula: $Zr_6O_{6+x}(BDC)_{6-x}$. Defect concentration is calculated as $x/(6-x)$.

Supplementary Table 1. The calculated ^{31}P chemical shifts of different coordination structures of TMP in defective UiO-66.

**TMP adsorption type**	**Cal. δ(^{31}P)/ppm**	**Exp. δ(^{31}P)/ppm**
Physical adsorption	-61 ~ -67	-63
Lewis acid sites	-32 ~ -36	-34
Brønsted acid sites	Listed bellow	-2 ~ -3
**$TMPH^+$ coordination site**	**Defect structure**	**Cal. δ(^{31}P)/ppm**
-COO	-Ac, $-OH_2$	-2.0 ~ -3.9
μ-O	-Ac, $-OH_2$	-0.4 ~ -3.6
$-OH_2$	-Ac, $-OH_2$	-1.3 ~ -3.5
μ-O	-OH, $-OH_2$	-2.2 ~ -4.3
$-OH_2$	-OH, $-OH_2$	-1.3 ~ -4.2
**TMP coordination site**	**Defect structure**	**Cal. δ(^{31}P)/ppm**
μ-OH	-OH, $-OH_2$	-6 ~ -12
-OH	-OH, $-OH_2$	-11 ~ -44
$-OH_2$	-OH, $-OH_2$	-21 ~ -23
μ-OH	-Ac, $-OH_2$	-15 ~ -20
$-OH_2$	-Ac, $-OH_2$	-14 ~ -26
μ-OH	-BDC-	-22 ~ -25

Supplementary Table 2. The calculated ^{31}P chemical shifts of different coordination structures of TMPO in defective UiO-66.

**TMPO coordination site**	**Defect structure**	**Cal. δ(^{31}P)/ppm**	**Exp. δ(^{31}P)/ppm**	**Species**
Under-coordinated Zr	Zr, -Ac	63	62	A
μ-OH	-BDC-	57 ~ 60	58	B
μ-OH	-Ac, $-OH_2$	55 ~ 57	55	C
$Zr-OH_2$	-Ac, $-OH_2$	54 ~ 55	53	D
Two molecules in the large cage		43 ~ 46	46	E
Single molecule in the large cage		40 ~ 44	42	F
"Head exposed" in the small cage		34 ~ 37	36	G
"Head hidden" in the small cage		30 ~ 34	32	H

Supplementary Table 3. The amount of total adsorbed TMPO and defect-associated TMPO in defective UiO-66 of different defect concentrations. The numbers are normalized by molar concentration of BDC.

Defect concentration by TGA	Total adsorbed TMPO	Defect-associated Species C+D
0.23	0.92	0.12
0.24	0.87	0.23
0.25	0.88	0.15
0.26	0.90	0.25
0.27	0.76	0.32
0.28	0.94	0.26
0.31	0.91	0.44

Supplementary References

1. M. J. Frisch H. B. Schlegel, G. E. Scuseria, G. W. T. et al. Gaussian 09, Revision E.01. Gaussian, Inc., Wallingford CT (2013).
2. Ditchfield, R. Self-consistent perturbation theory of diamagnetism. Mol. Phys. 27, 789–807 (1974).
3. Friedrich, K., Seifert, G. & Großmann, G. Nuclear magnetic shielding in molecules. The application of GIAO's in LCAO-Xα-calculations. Zeitschrift für Phys. D Atoms, Mol. Clust. 17, 45–46 (1990).
4. Van Der Spoel, D. et al. GROMACS: Fast, flexible, and free. J. Comput. Chem. 26, 1701–1718 (2005).
5. Pronk, S. et al. GROMACS 4.5: A high-throughput and highly parallel open source molecular simulation toolkit. Bioinformatics 29, 845–854 (2013).
6. Abraham, M. J. et al. Gromacs: High performance molecular simulations through multi-level parallelism from laptops to supercomputers. SoftwareX 1–2, 19–25 (2015).
7. Yang, Q. et al. Functionalizing porous zirconium terephthalate UiO-66(Zr) for natural gas upgrading: A computational exploration. Chem. Commun. 47, 9603–9605 (2011).
8. Wang, J., Wolf, R. M., Caldwell, J. W., Kollman, P. A. & Case, D. A. Development and testing of a general amber force field. J. Comput. Chem. 25, 1157–1174 (2004).
9. Wang, J., Wang, W., Kollman, P. A. & Case, D. A. Automatic atom type and bond type perception in molecular mechanical calculations. J. Mol. Graph. Model. 25, 247–260 (2006).
10. Jorgensen, W. L., Chandrasekhar, J., Madura, J. D., Impey, R. W. & Klein, M. L. Comparison of simple potential functions for simulating liquid water. J. Chem. Phys. 79, 926–935 (1983).
11. Hess, B., Bekker, H., Berendsen, H. J. C. & Fraaije, J. G. E. M. LINCS: A linear constraint solver for molecular simulations. J. Comput. Chem. 18, 1463–1472 (1997).
12. Darden, T., York, D. & Pedersen, L. Particle mesh Ewald: An N·log(N) method for Ewald sums in large systems. J. Chem. Phys. 98, 10089–10092 (1993).
13. Evans, D. J. & Holian, B. L. The Nose-Hoover thermostat. J. Chem. Phys. 83, 4069–4074 (1985).

14. Fu, Y. *et al.* Defect-Assisted Loading and Docking Conformations of Pharmaceuticals in Metal–Organic Frameworks. *Angew. Chemie Int. Ed.* **60**, 7719–7727 (2021)
