# Supplementary material for: Molecular identification and quantification of defect sites in metal-organic frameworks with NMR probe molecules
Source: Nat Commun. 2022 Aug 30;13:5112. doi: 10.1038/s41467-022-32809-9 (PMC9427814; doi:10.1038/s41467-022-32809-9)
Supplement: Supplementary file 2 — Description of Additional Supplementary Files [file 41467_2022_32809_MOESM2_ESM.docx]

**Description of Additional Supplementary Files**

File Name: Supplementary data 1

Description: TMP adsorbed on μ-OH in defective UiO-66 with acetate and water

File Name: Supplementary data 2

Description: TMP adsorbed on hydroxyl in defective UiO-66 with hydroxyl and water

File Name: Supplementary data 3

Description: TMPO adsorbed on hydroxyl in defective UiO-66 with hydroxyl and water

File Name: Supplementary data 4

Description: TMPO adsorbed on μ-OH in defective UiO-66 with acetate and water

File Name: Supplementary data 5

Description: TMPO adsorbed on μ-OH in defective UiO-66 with formate

File Name: Supplementary data 6

Description: TMP adsorbed on μ-OH in defective UiO-66 with hydroxyl and water

File Name: Supplementary data 7

Description: TMPO adsorbed on μ-OH in defective UiO-66 with hydroxyl and water

File Name: Supplementary data 8

Description: TMP adsorbed on μ-OH in ideal UiO-66

File Name: Supplementary data 9

Description: TMPO adsorbed on μ-OH in ideal UiO-66

File Name: Supplementary data 10

Description: TMP adsorbed on water in defective UiO-66 with acetate and water

File Name: Supplementary data 11

Description: TMPO adsorbed on water in defective UiO-66 with acetate and water

File Name: Supplementary data 12

Description: TMP adsorbed on water in defective UiO-66 with hydroxyl and water

File Name: Supplementary data 13

Description: TMPO adsorbed on water in defective UiO-66 with hydroxyl and water

File Name: Supplementary data 14

Description: TMP adsorbed on Zr in defective UiO-66 with acetate

File Name: Supplementary data 15

Description: TMPO adsorbed on Zr in defective UiO-66 with acetate

File Name: Supplementary data 16

Description: TMP adsorbed on Zr in defective UiO-66 with hydroxyl

File Name: Supplementary data 17

Description: TMPO adsorbed on Zr in defective UiO-66 with hydroxyl

File Name: Supplementary data 18

Description: TMP adsorbed on Zr in defective UiO-66 with water

File Name: Supplementary data 19

Description: TMPO adsorbed on Zr in defective UiO-66 with water
